# Dr. Nathaniel Pendleton Dandridge

**Published:** 1910-12

**Authors:** 


Dr. Nathaniel Pendleton Dandridge, of Cincinnati, died at his home November 6, 1910, aged 64 years. Dr. Dandridge was for many years one of the leading surgeons of the southwest, taught surgery in the Miami Medical College and was dean of the faculty for many years, was surgeon to the Cincinnati hospital of which he was a director in the board, and was prominent in medical society work. He was a Fellow of the American Surgical Association, and a man of high social standing. He graduated in medicine at the College of Physicians and Surgeons at New York in 1870.

